# Case Report: An endogenous endophthalmitis case caused by *Haemophilus influenzae*

**DOI:** 10.3389/fmed.2025.1692870

**Published:** 2025-10-27

**Authors:** Chia Hsuan Tsai, Li-Teh Liu, En Chi Hsu, Shao-Yu Sung, Jih-Jin Tsai

**Affiliations:** ^1^Department of Clinical Education and Training, Kaohsiung Medical University Hospital, Kaohsiung City, Taiwan; ^2^Department of Medical Laboratory Science and Biotechnology, College of Medical Technology, Chung Hwa University of Medical Technology, Tainan, Taiwan; ^3^Department of Internal Medicine, Kaohsiung Medical University Hospital, Kaohsiung City, Taiwan; ^4^Department of Ophthalmology, Kaohsiung Medical University Hospital, Kaohsiung City, Taiwan; ^5^Tropical Medicine Center, Kaohsiung Medical University Hospital, Kaohsiung City, Taiwan; ^6^Division of Infectious Diseases, Department of Internal Medicine, Kaohsiung Medical University Hospital, Kaohsiung City, Taiwan; ^7^School of Medicine, College of Medicine, Kaohsiung Medical University, Kaohsiung City, Taiwan

**Keywords:** endogenous endophthalmitis, *haemophilus influenzae*, intravitreal injection, oral cancer, Behçet disease

## Abstract

*Haemophilus influenzae* is a bacterium that typically colonizes the human respiratory tract. However, it can also infect the eyes, potentially leading to endophthalmitis. The general prognosis of endophthalmitis is often poor, frequently resulting in decreased visual acuity. We present the case of a 71-year-old male who experienced sudden blurring of vision, pain, and a significant hypopyon in the fundus of his right eye resulting from *Haemophilus influenzae*. According to previous reports, many cases of *Haemophilus influenzae* endophthalmitis have poor outcomes, often resulting in permanent visual impairment. However, the patient achieved an excellent prognosis in our case due to prompt diagnosis and emergent treatment.

## Introduction

*Haemophilus influenzae* (*H. influenzae*) typically colonizes the human respiratory tract and is known to cause meningitis and epiglottitis in children and pneumonia in adults. However, *H. influenzae* can occasionally colonize the eyes. This bacterium can be classified into encapsulated and unencapsulated strains, with encapsulated strains being more virulent. It is primarily transmitted through airborne droplets or direct contact with secretions from infected individuals (1).

Endophthalmitis is an inflammatory condition of the intraocular fluids, including the vitreous and aqueous humor. It is categorized into three types: postoperative endophthalmitis, post-traumatic endophthalmitis, and endogenous endophthalmitis (EE). EE accounts for approximately 2 to 8% of all cases of endophthalmitis.

Without timely diagnosis and treatment, endophthalmitis can lead to severe complications and poor visual outcomes. Prompt treatment is, therefore, essential for visual recovery. Studies have shown that EE primarily occurs in immunocompromised individuals, such as those with diabetes or suppressed immune systems (2). Common EE-associated pathogens include *Staphylococcus aureus, Streptococcus pneumoniae, Klebsiella pneumoniae, Candida albicans*, and Aspergillus. However, *H. influenzae* is a rare causative agent of EE (2).

According to previous literature, the prognosis of *H. influenzae* endophthalmitis is generally poor (2). However, in the case we present, the outcome was remarkably favorable. The patient showed no conjunctival congestion, a clear cornea, and no hypopyon in the anterior chamber. Most notably, the patient reported that his visual acuity returned to its pre-illness baseline.

## Case report

**Figure 1:**
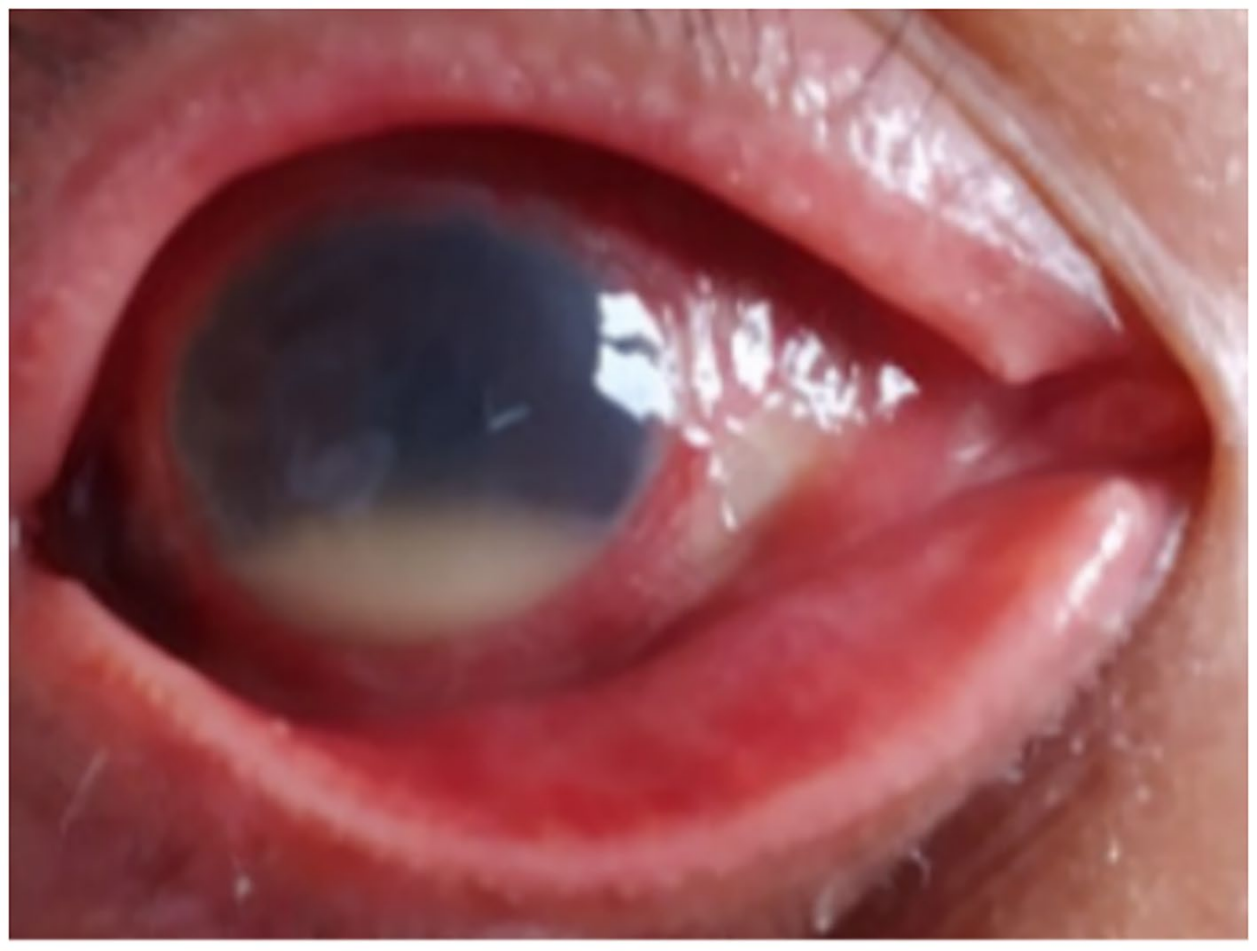
PSO 1 day. Hypopyon in the right eye. *PSO: post-symptom onset.

We present the case of a 71-year-old man who experienced a sudden onset of blurred vision and mild pain in his right eye. He had no recent history of ocular surgery or trauma. Ocular examination revealed hypopyon in the right eye (Figure 1). His underlying medical conditions included oral cancer, Behçet’s disease, and benign prostatic hyperplasia. For the management of his Behçet’s disease, the patient was receiving Leflunomide (20 mg daily), a disease-modifying antirheumatic drug (DMARD).

Upon arrival at the emergency department (ED), his vital signs were stable: blood pressure of 157/79 mmHg, heart rate of 96 beats per minute, respiratory rate of 20 breaths per minute, body temperature of 37.1 °C, and oxygen saturation (SpO₂) of 98%. Laboratory tests showed no leukocytosis or elevated C-reactive protein (CRP).

A slit-lamp ophthalmic examination of the right eye revealed the following: Best corrected visual acuity (BCVA) of hand movement (HM) at 1meter, IOP (intraocular pressure) of 35 mmHg, congested conjunctiva, mild corneal edema with diffuse fine keratic precipitates (KPs), the cell grade of the anterior chamber of 3+, the grade of aqueous flare of 1 + and the fundus being veiled due to vitreous opacity. Blood culture results confirmed *Haemophilus influenzae*.

In the ED, the patient was prescribed Combigan (brimonidine tartrate and timolol maleate) (twice daily), Econopred (prednisolone acetate 1% per ml) (every 3 h), and Levofloxacin (5 mg/mL) (every 2 h) eye drops. He received an immediate intravitreal injection (IVI) of vancomycin, ceftazidime, and dexamethasone, with a repeat IVI administered three days later and three additional weekly injections.

In the infection ward, initial systemic antibiotic treatment with Cefoperazone (1 g every 12 h) and Sulbactam (1 g every 12 h) was later switched to ceftriaxone (2 g every 12 h) after being confirmed as *H. influenzae*.

Extensive investigations were performed to identify the primary infection focus. The patient remained afebrile during his hospital stay. Abdominal CT, cardiac echocardiography, and brain CT angiography showed no abscess, vegetation, valvular disease, or intracranial lesion. Tumor marker screening revealed no evidence of metastasis or malignancy contributing to the vision defect.

**Figure 2:**
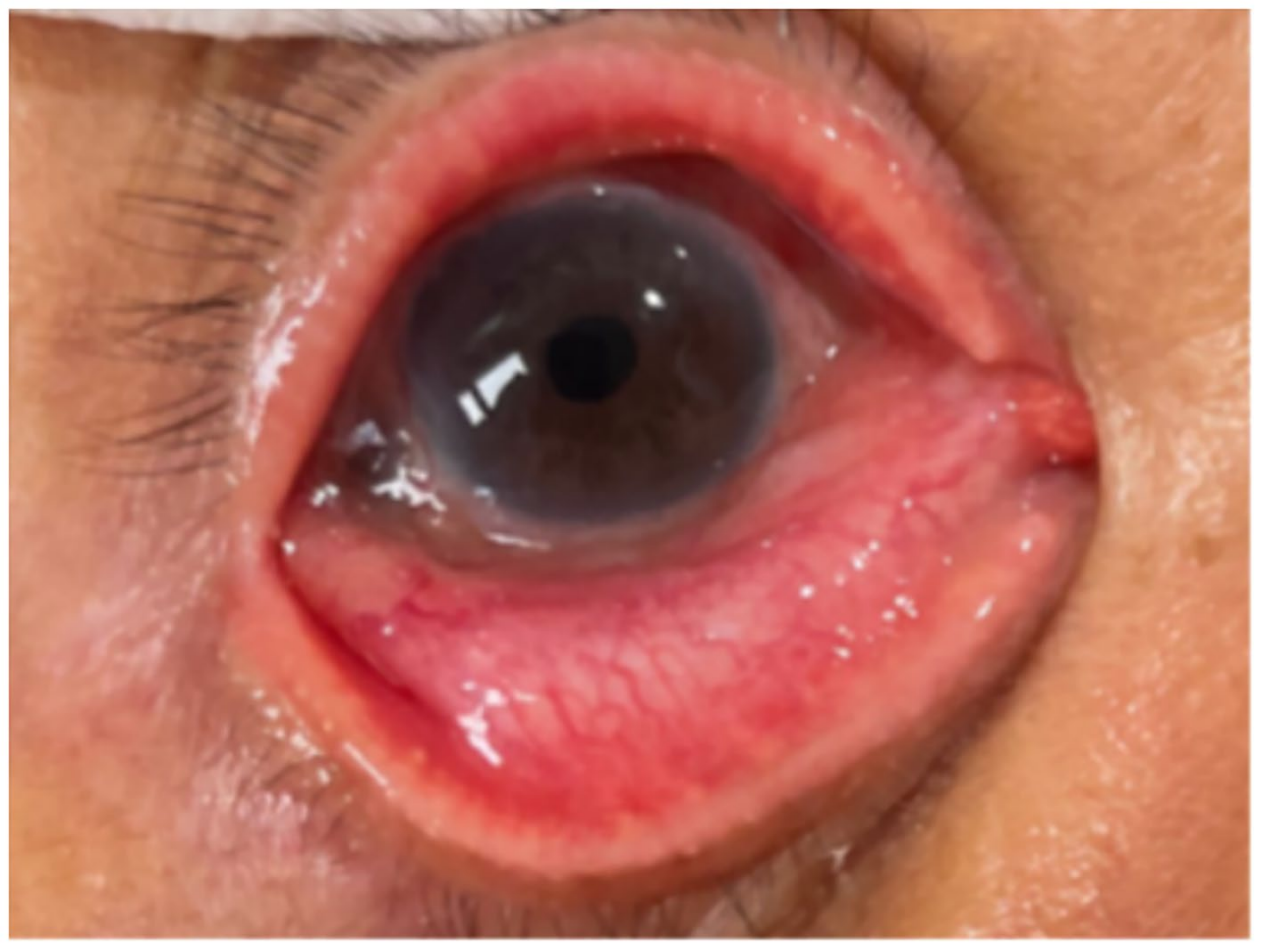
PSO 25 days. Resolution of hypopyon. *PSO: post-symptom onset.

After approximately four weeks of ceftriaxone treatment and ocular treatments, the patient’s BCVA improved to 0.1. This was a significant improvement from hand movement at 1 meter upon presentation, and the patient considered this outcome to be a restoration of his functional baseline vision. The patient showed no conjunctival congestion, a clear cornea, and no hypopyon in the anterior chamber (Figure 2). Following this, his topical regimen was adjusted to include Sinomin (sulfamethoxazole 4%) eye drops for two months and Flucason (fluorometholone 0.1%) eye drops for three months, which were gradually tapered. His intraocular pressure (IOP) was well-controlled with the continuous use of Timoptol XE (timolol maleate 0.5%) eye drops. The patient’s condition, including visual acuity and IOP, remained stable throughout the five-month follow-up period.

## Discussion

While intraocular fluid culture is the diagnostic gold standard for endophthalmitis, this procedure was deferred. Given the patient’s positive blood culture clearly identifying *H. influenzae* and the critical need to preserve vision, the clinical team prioritized immediate therapeutic intervention over the procedural risks of a vitreous tap or biopsy. Similarly, further characterization of the bacterial strain through biotyping or serotyping was not performed, as this is not standard procedure in our clinical laboratory. These factors represent limitations in our case report.

Our patient, who was immunocompromised due to squamous cell carcinoma and Behçet’s disease, was at increased risk for endophthalmitis and possibly had poor outcomes. Nevertheless, he remarkably recovered due to timely and appropriate treatments, including intravitreal injections and systemic antibiotics.

Endogenous endophthalmitis is a rare but serious condition, accounting for only 5–15% of all endophthalmitis cases. *H. influenzae* is an uncommon cause of endogenous endophthalmitis (2).

We summarized the 23 *H. influenzae*-related endogenous endophthalmitis cases, including the four cases reported by A. Gupta (3). One case worsened to a visual acuity (VA) 4/60, two cases required evisceration, and one case progressed to enucleation. However, the report did not mention the patient’s background. We organized 19 cases in Table 1, including detailed patient backgrounds (1, 4–6). Most of them (17/19) were immunocompetent, and only two (2/19) of them resolved (1, 5). Overall, nearly 90% resulted in poor visual outcomes. The Endophthalmitis Vitrectomy Study (EVS), focusing on the management of endophthalmitis, did not include data on *H. influenzae* (3). No evidence to support aggressive treatment for endophthalmitis caused *by H. influenza.* Therefore, close monitoring and clinical judgment remain the most important considerations (3).

**Table 1:** *Haemophilus influenzae*-related endogenous endophthalmitis cases from the literature.

Case, reference, year published	Age, sex	Immune status	Clinical presentation	outcomes
1. Boomla and Quilliam (1981) (1)	20-month-old, F	Healthy	Red right eye, hazy cornea without ulceration, and hypopyon obscuring the fundus	Resolved with subconjunctival gentamicin and systemic ampicillin, cloxacillin, and gentamicin.
2. Yoder et al. (2004) (4)	16 patients, median age of 68 years (range, 6 months–83 years)	Healthy	post-trabeculectomy (*n* = 7), post–cataract surgery (*n* = 6), post–pars plana vitrectomy (*n* = 1), post–secondary intraocular lens insertion (*n* = 1), Post-suture removal (extracapsular cataract wound) (*n* = 1)	Poor visual outcomes despite prompt intravitreal antibiotic treatment, which was effective against the organisms.
3. Haruta et al. (2017) (5)	1-year-old, F	Hyposplenism	Cloudy cornea in the right eye, IP = 43 mmHg, 8-day fever.	Resolved with intravenous meropenem/vancomycin, topical gatifloxacin/cefmenoxime drops, and vitrectomy. VA was 20/40.
4. Tabuenca Del Barrio et al. (2021) (6)	13-year-old, M	immunocompetent	right eye pain with floaters and decreased VA	Poor visual outcome

VA: Visual Acuity, IP: intraocular pressure, F: Female, M: Male.

A key aspect of our case is the favorable outcome, which contrasts with the poor prognosis reported in nearly 90% of previous cases. Our therapeutic strategy, which involved a total of five intravitreal injections and a prolonged four-week course of systemic ceftriaxone, represents an aggressive management approach. This intensive regimen may be a key factor in our patient’s successful visual recovery and could serve as a potential model for future cases. The issue of antibiotic resistance is a growing concern in ophthalmology. As noted, *H. influenzae* strains, particularly non-typeable strains (NTHi), increasingly exhibit beta-lactamase production and resistance to multiple drug classes. This underscores the importance of appropriate antibiotic selection and stewardship. In light of rising resistance, exploring alternative or adjunctive therapies is warranted. For instance, the use of broad-spectrum antiseptics is gaining attention as a therapeutic approach due to their nonselective mechanisms of action, which may circumvent conventional resistance pathways (7). While not applied in our case, such strategies may become increasingly important in managing ocular infections.

Regarding the reason why *H. influenzae* infects human eyes, it is associated with systemic infection, and bacteria may spread from the infection focus to the eyes through the bloodstream. The source of the infection may also come from the upper respiratory tract (8). Endogenous bacteria can cause endophthalmitis by crossing the blood-ocular barrier of the eyes, originating from an infection focus in distant organs. Infection sources may come from endocarditis, meningitis, or urinary tract infection. In our patient, extensive investigations including abdominal CT, cardiac echocardiography, and brain CT angiography were performed, but they did not reveal a definitive primary focus of infection. However, considering that the upper respiratory tract is the natural reservoir for *H. influenzae*, a subclinical infection or colonization at this site represents the most likely portal of entry for hematogenous spread. Besides, patients with immune deficiency are more prone to endophthalmitis, such as diabetes mellitus (DM), cancer, use of immunosuppressive medication, or old age (9). However, an immunocompetent host can still develop endophthalmitis (6).

Regarding the new insights on *H. influenzae*, it is classified into eight biotypes and six serotypes. Biotypes and serotypes may determine the patterns of colonization of *H. influenzae* and influence the severity of the infection. Alrawi AM group found that endophthalmitis is mainly caused by Biotype II of *H. influenzae* (8). Recent studies found that biotype II of non-encapsulated *H. influenzae* covered most ocular *H. influenzae* isolates. However, recent studies could not conclude the direct relationship between *H. influenzae* subtype and location of ocular infection. The spleen plays a crucial role in eliminating encapsulated bacteria like *H. influenzae*. Patients with hyposplenism may be prone to bloodstream infection of *H. influenzae* and endogenous endophthalmitis (5).

For the current antibiotic resistance status of *H. influenzae*, many strains produce beta-lactamase enzymes to resist ampicillin. There are also minor strains that belong to non-beta-lactam-resistance types, showing resistance to macrolides, fluoroquinolones, tetracyclines, and trimethoprim-sulfamethoxazole (10). Since the introduction of the *H. influenzae* type b (Hib) vaccine in 1985 in the United States and 1996 in Taiwan, the Hib infection rate has gradually decreased, and the younger generation is now infected mainly by non-typeable *H. influenzae* (NTHi) (8). NTHi strains often exhibit increased beta-lactam resistance due to their production of beta-lactamase. Besides, the rise of multidrug-resistant (MDR) (NTHi) is becoming an increasingly serious public health concern in Taiwan (11).

Many studies showed that endophthalmitis resulting from *H. influenzae* infection typically leads to poor visual outcomes even with prompt antibiotic treatment and often requires vitrectomy (6). Endophthalmitis resulting from *H. influenzae* may not lead to death, but endophthalmitis caused by filamentous fungi may be more prone to causing death (12). Recently, medical management consists of intravitreal antibiotics, systemic antibiotics, topical and subconjunctival antibiotics, corticosteroids, and surgical management. Our case, even in the immunocompromised conditions, presented with a good clinical outcome under appropriate and aggressive treatments. The best way to prevent a worse outcome in endophthalmitis caused by *H. influenzae* may be to use the proper treatment promptly, carefully monitor the patient’s condition, and adjust the treatment accordingly.

## Data Availability

The original contributions presented in the study are included in the article/supplementary material, further inquiries can be directed to the corresponding author.
